# Regulation of iron metabolism and ferroptosis in cancer stem cells

**DOI:** 10.3389/fonc.2023.1251561

**Published:** 2023-09-01

**Authors:** Hailiang Wang, Zhongyan Zhang, Shiye Ruan, Qian Yan, Yubin Chen, Jinwei Cui, Xinjian Wang, Shanzhou Huang, Baohua Hou

**Affiliations:** ^1^Department of Hepatobiliary Surgery, Weihai Central Hospital Affiliated to Qingdao University, Weihai, China; ^2^Department of General Surgery, Guangdong Provincial People’s Hospital (Guangdong Academy of Medical Sciences), Southern Medical University, Guangzhou, China; ^3^The Second School of Clinical Medicine, Southern Medical University, Guangzhou, China; ^4^Department of General Surgery, Heyuan People’s Hospital, Heyuan, China; ^5^Department of General Surgery, South China University of Technology School of Medicine, Guangzhou, China

**Keywords:** CSCs, ferroptosis, iron metabolism, lipid peroxidation, GPX4-GSH, FSP1-CoQ10

## Abstract

The ability of cancer stem cells (CSCs) to self-renew, differentiate, and generate new tumors is a significant contributor to drug resistance, relapse, and metastasis. Therefore, the targeting of CSCs for treatment is particularly important. Recent studies have demonstrated that CSCs are more susceptible to ferroptosis than non-CSCs, indicating that this could be an effective strategy for treating tumors. Ferroptosis is a type of programmed cell death that results from the accumulation of lipid peroxides caused by intracellular iron-mediated processes. CSCs exhibit different molecular characteristics related to iron and lipid metabolism. This study reviews the alterations in iron metabolism, lipid peroxidation, and lipid peroxide scavenging in CSCs, their impact on ferroptosis, and the regulatory mechanisms underlying iron metabolism and ferroptosis. Potential treatment strategies and novel compounds targeting CSC by inducing ferroptosis are also discussed.

## 1 Introduction

Despite significant advancements in cancer prevention, diagnosis, and treatment in recent years, the global cancer burden remains significant. Cancer treatment remains a significant challenge, particularly addressing cancer progression, recurrence, drug resistance, and metastasis, which are associated with poor prognosis (1). Ample evidence indicates that cancer stem cells (CSCs) play a crucial role in these processes (2). The presence of a small fraction of CSCs in tumor tissue, which have the ability to self-renew, differentiate, and generate new tumors, is the termed the CSC hypothesis (3).

### 1.1 Cancer stem cell hypothesis

Although the true mechanisms of tumorigenesis are still not fully understood, the cancer stem cell hypothesis is well suited to explain tumor progression, drug resistance, metastasis, and recurrence and continues to be supported by experimental results and clinical phenomena. First, CSCs are often associated with poor prognosis. One study confirmed the high expression of biomarkers of CSCs was strongly associated with significantly lower overall and/or disease-free survival in patients with a variety of cancers in 82% of 234 reported survival analyses (4), such as CD133 (5), CD44 (6), ALDH (4), OCT-4, and Nanog (7). In addition, overexpression of ATP-binding box (ABC) efflux transporters in CSCs promotes tumor resistance through a drug efflux mechanism, resulting in chemotherapy failure (8). Secondly, interactions between CSCs and their niche maintain self-renewal and promote drug resistance, metastasis, and relapse. Many studies have shown that CSCs visibly alter iron metabolism (9), lipid metabolism (10), multiple cell signaling (11), tumor microenvironment (12, 13), redox regulation (14, 15), epithelial-mesenchymal transformation (EMT) (7, 16), and other aspects. In addition, due to their plasticity, quiescent CSCs can transform into cycling CSCs, leading to metastasis and relapse (17). Thirdly, quiescent CSCs may be the root cause of the difficulty in eradicating tumors. Telomeres and telomerase are known to play an important role in human aging and cancer, maintaining genomic stability and being critical for cell proliferation (18). Using simultaneous single-cell analysis of the tranome and telomeres, researchers found that CSCs in the quiescent state have low telomerase activity and short telomeres, low cell proliferation, but higher stemness and can mutate into tumor epithelial cells that express telomerase and acquire longer telomeres into a cell proliferative state, which may be the underlying cause of tumor recurrence and drug resistance (19).

Therefore, many experts believe that therapeutic strategies targeting CSCs have great potential, and that there is a need to investigate drugs that target CSCs to improve outcomes (20). However, to date, no drugs targeting CSCs have been approved for clinical use (11), and it may be combining these strategies or drugs targeting CSCs with other antitumor treatments that could lead to a better prognosis.

### 1.2 Ferroptosis is the hope for tumor treatment

Ferroptosis is a form of iron-mediated programmed cell death (21, 22) caused by the disruption of cell membranes due to excessive peroxidation of phospholipids containing polyunsaturated fatty acids (PUFAs) and does not exhibit apoptotic features. In cells, the biological activity of iron is mainly reflected in the electron transfer between trivalent iron (Fe^3+^) and ferrous iron (Fe^2+^) in various physiological reactions in which Fe^2+^ reacts non-enzymatically with H_2_O_2_ to produce hydroxyl radicals that can oxidize PUFAs in the cell membrane; this is also known as the Fenton reaction (23, 24). As the concentration of lipid peroxides gradually increases, the stability of the cell membrane is compromised, and ferroptosis occurs when the capacity of the intracellular redox system to remove lipid peroxides is exceeded.

Ferroptosis has gradually come into focus in tumor treatment research. Although our understanding of ferroptosis is not yet complete, as studies have accumulated, we have found that ferroptosis is closely related to tumor progression, treatment, and prognosis. The first is the predictive role of ferroptosis-associated genes. By integrating the expression of ferroptosis genes in bladder cancer patients from The Cancer Genome Atlas (TCGA) and the Gene Expression Omnibus (GEO) databases with clinical data, one study found that the expression levels of ferroptosis-related genes (*SLC7A11, GPX4, ACSL4*, etc.) could be used to predict tumor progression and prognosis (25). Secondly, ferroptosis affects drug sensitivity. Huang et al. found that activation of the PI3K/AKT/NRF2 axis in sorafenib-resistant advanced hepatocellular carcinoma cells significantly upregulated ABCC5 expression, while stabilizing solute carrier family 7 member 11 (SLC7A11) protein and increasing intracellular glutathione (GSH) content, thereby inhibiting ferroptosis (26). Platinum resistance is more likely to develop in lung cancer brain metastases through a mechanism that reduces ferroptosis by upregulating glutathione peroxidase 4 (GPX4) expression and inhibiting GSH depletion via the Wnt/NRF2/GPX4 axis (27). Therefore, the inhibition of ferroptosis can reduce tumor sensitivity to drugs and, hence, drug resistance. Similarly, the induction of ferroptosis increases drug sensitivity. In studies on various tumors such as head and neck cancer (28), pancreatic cancer (29), hepatocellular carcinoma (30), and bladder cancer (31), the induction of ferroptosis through mechanisms such as the regulation of GSH metabolism, lipid metabolism, and redox was found to be an effective strategy for overcoming drug resistance. Ferroptosis is an effective therapeutic approach for targeting CSCs. There are *in vivo* and *in vitro* studies demonstrating that MiR-375 can reduce the stemness of gastric CSCs by targeting SLC7A11 to induce ferroptosis (32). Vitamin D (33) and erastin (34) specifically inhibit SLC7A11 to induce ferroptosis and suppress the proliferation and sphere-forming ability of colorectal CSCs. Notably, colorectal CSCs have also been found to be more sensitive to ferroptosis (34). Similar phenomena have been observed in other tumors; glioma stem cells with high aldehyde dehydrogenase isoform 1 (ALDH1) expression, which are highly resistant to standard treatment regimens, are sensitive to ferroptosis induced by the ferroptosis inducer RSL3, and their sensitivity increases with the expression level of ALDH1 (35). These results suggest that the induction of ferroptosis in CSCs or a combination of conventional antitumor therapies is a promising therapeutic strategy for tumor eradication. As mentioned earlier, the process of ferroptosis can be summarized in three aspects: 1. An increase in the labile ferrous iron content 2. accumulation of phospholipid peroxides; and 3. Inadequate scavenging of lipid peroxides. This review summarizes the current understanding of ferroptosis in CSCs by analyzing these three aspects.

## 2 Iron metabolism in CSCs

Iron is an essential component of the body that is required for cell metabolism and proliferation, particularly in tumor cells. In contrast to normal cells, rapidly proliferating tumor cells have altered iron levels and the expression and function of iron metabolism-related proteins, which in turn affect many physiological processes, including DNA synthesis and repair, cell cycle regulation, angiogenesis, metastasis, tumor microenvironment, metabolic reprogramming, and epigenetic regulation (23, 36). Iron metabolism plays a key role in tumor cell survival, making it a popular topic in antitumor therapy research in recent years. While tumor cells have high iron levels because of their need for rapid proliferation (36), CSCs accumulate more iron than non-CSCs, and iron metabolism affects iron homeostasis in CSCs in terms of iron uptake, storage, and transport (24, 37).

### 2.1 Promote iron uptake in CSCs

Iron uptake by CSCs was significantly enhanced. On the one hand, extracellular stable Fe^3+^ enters the cytoplasm by binding to transferrin (TF) and then complexes with the transferrin receptor (TFR), followed by endocytosis. Compared with non-CSCs, endocytosis was significantly enhanced in CSCs. Researchers have found through ‘iron tracer’ experiments that glioblastoma stem cells can take up iron from the extracellular space more efficiently than other tumor cells, and that two of the key links are TFR and ferritin (37), and it is true that CSCs express higher levels of TFRs and ferritin than other tumor cells. The same situation appears in studies on iron metabolism in breast and ovarian CSCs. In these two studies, CSCs were found to express higher levels of TFRs than non-CSCs (38, 39), and high levels of intracellular iron were consistent with high levels of TFR expression (39). In contrast, CSCs increased iron uptake through CD44-mediated iron endocytosis. In general, tumor cells tend to acquire stemness during EMT. CD44-mediated endocytosis of iron-binding hyaluronan has been observed in primary tumor cells, which have an increased need for iron during EMT and increase iron uptake by upregulating CD44-mediated endocytosis (40).

However, the CSCs did not promote iron uptake. CD133, a CSC stemness marker, is a negative regulator of iron uptake and inhibits TR-mediated iron endocytosis (41). As the researchers did not test intracellular iron levels or other indicators of ferroptosis in their experiments, the extent to which the inhibition of iron uptake by CD133 affects intracellular iron levels and ferroptosis is unclear. We suggest that the effects of stemness genes in CSCs on the positive and negative regulatory mechanisms of iron uptake are not contradictory and that CSCs may adopt regulatory mechanisms that are beneficial to themselves at different times to maintain high levels and homeostasis of intracellular iron, which is worth investigating.

### 2.2 Maintain homeostasis of the labile iron pool

Extracellular Fe^3+^ enters the cytosol via endocytosis and is reduced to Fe^2+^ by the six-transmembrane epithelial antigen of prostate 3 (STEAP3) in the acidic environment of the endosome. Divalent metal transporter protein 1 (DMT1) releases ferrous iron from lysosomes into the cytoplasm, where it joins the labile iron pool (LIP) (42). This pathway, by which extracellular iron uptake occurs, affects homeostasis of the labile iron pool. In a study on ferroptosis-related gene signatures associated with prognosis in neuroblastoma, STEAP3 was found to be highly expressed (43), and increased STEAP3 activity undoubtedly increased the amount of labile iron. Another study found that higher levels of STEAP3 expression in gliomas were associated with worse prognosis for patients and promoted the proliferation and self-renewal of glioma stem cells (44). Similarly, the role of DMT1 is critical, it was found that DMT1 inhibitors can lead to the accumulation of Fe^2+^ in lysosomes, induce ferroptosis-like disruption of the lysosomal membrane, release large amounts of Fe^2+^ into the cytoplasm, and kill CSCs (45). Similarly, The expression of DMT1 mRNA and protein was significantly increased in aggressive glioblastoma cells treated with temozolomide, which led to an increase in intracellular iron content and induced ferroptosis (46). In ovarian cancer, high DMT1 expression was associated with poor overall patient survival (47).

Although the effect of extracellular iron uptake on LIP for intracellular stores, LIP homeostasis may be maintained by nuclear receptor coactivator 4 (NCOA4)-mediated ferritin autophagy. However, the function of NCOA4 in CSCs remains unclear. Recently, tripartite motif-containing protein 7 (TRIM7) was found to be highly expressed in human aggressive glioblastoma cells and to inhibit ferritin autophagy, reduce Fe^2+^ levels in glioblastoma cells, and suppress ferroptosis by ubiquitinating NCOA4 (48). Similarly, the induction of ferritin autophagy mediated by NCOA4 increases the sensitivity of aggressive glioblastoma cells to ferroptosis (49). However, in a specific study, researchers evaluated the effect of long-term moderate-intensity static magnetic field (SMF) on osteosarcoma stem cells and found that exposure to SMF-activated NCOA4, induced ferritin autophagy, increased intracellular Fe^2+^ levels, and promoted the self-renewal ability of osteosarcoma stem cells (50). Concerning these inconsistent results, we hypothesize that NCOA4’s effect on CSCs could rely on the base Fe^2+^ level in LIP, which is sustained within certain limits. If the Fe^2+^ basal level in LIP reaches the upper limit (critical state for triggering ferroptosis), increased NCOA4 expression could heighten the Fe^2+^ level, potentially stimulating ferroptosis and inhibiting stemness. Conversely, if the Fe^2+^ basal level in LIP is at the lower limit, up-regulating NCOA4 expression could boost stemness by enhancing the Fe^2+^ level in LIP. We deem our conjecture meriting further investigation.

### 2.3 Increased iron storage in CSCs

Excess intracellular iron is stored in ferritin, a type of ferritin consisting of two protein subunits, a heavy chain (FtH) and a light chain (FtL), which can store more than 4,000 iron atoms. Ferritin has ferrous oxidase activity (51) and can oxidize Fe^2+^ to inert Fe^3+^ and bind to it, thereby preventing the Fenton reaction. When Fe^2+^ levels in LIP decrease, NCOA4 mediates ferritin phagocytosis, releasing Fe^2+^ into the cytoplasm and maintaining LIP homeostasis (51).

High ferritin levels are closely associated with CSC stemness. The cytokine oncostatin M (OSM) is highly expressed in breast CSCs and increases ferritin levels in stem cells, whereas the knockdown of ferritin expression impairs the ability of OSM to induce stemness (39). Similarly, ferritin knockdown inhibits glioblastoma growth both *in vitro* and *in vivo* (37). Therefore, it has been suggested that intracellular ferritin content is a key factor in ferroptosis susceptibility, with more ferritin creating more Fe^3+^ stores and less Fe^2+^ in the LIP, resulting in greater ferroptosis resistance (52), whereas low ferritin knockdown releases more Fe^2+^ into the cell, resulting in ferroptosis susceptibility (53). However, there is another possibility. More ferritin implies more intracellular iron stores to meet the high iron demand of CSCs. No tumor formation was observed in mice inoculated with ferritin-knockdown glioblastoma cells (37). Furthermore, FtH can enter the nucleus, participate in DNA synthesis (23), and is a rate-limiting regulator of epigenetic plasticity (40). Therefore, FtH, with ferrous oxidase activity, can prevent damage to the nuclear membrane by reducing the level of Fe^2+^ in the nucleus during DNA synthesis. This is because the nuclear membrane remains intact during ferroptosis (42). Therefore, we speculate that the role of high ferritin expression in CSC ferroptosis involves two aspects. First, it increases intracellular iron reserves and plays an important role in maintaining high levels of intracellular Fe^2+^, followed by maintaining nuclear membrane stability by binding to Fe^2+^ in the nucleus.

However, another study contradicts this conclusion. Analysis of public microarray databases for ovarian cancer showed that low FtH expression is associated with poor prognosis. In subsequent experiments, FtH knockdown promoted EMT, pellet formation, and invasiveness of SKOV3 cells. Researchers found that FtH knockdown led to changes in the expression of the miRNA network, suggesting that ferritin may affect other pathways of tumor progression in addition to iron storage, which is why it affects tumor cell stemness (54). Unfortunately, the researchers did not measure iron levels in cells.

### 2.4 Suppression of iron export from CSCs

Ferroportin (FPN) transports excess intracellular iron out of the cell as Fe^3+^ and is regulated by hepcidin, which binds directly to FPN and inhibits its activity. Many studies have shown low FPN expression in various tumor cells. In breast cancer, FPN expression decreases, hepcidin expression increases, and intracellular Fe^2+^ levels increase, which is associated with poor prognosis and an invasive phenotype; Overexpression of FPN can inhibit tumor growth (55). Thus, the ferroportin–hepcidin axis plays an important role in regulating intracellular iron levels. Many CSCs exhibit such alterations. For example, FPN is transcriptionally downregulated in cholangiocarcinoma CSCs (56) and is hypoexpressed in ovarian cancer CSCs (38). When overexpressed, FPN inhibits EMT, cytokinesis, and colony formation in mouse breast cancer cells, preventing tumor growth and metastasis to the liver and lungs but does not cause significant cell death (57). Hepcidin is mainly synthesized by the liver and is involved in the regulation of systemic iron homeostasis. At the cellular level, tumor cells can produce hepcidin themselves, which negatively regulates intracellular iron (58). Breast cancer cells in spheroids have higher hepcidin expression and intracellular iron levels than cells grown in monolayers (59). Thus, hepcidin plays an important role in maintaining CSC stemness.

**Figure 1:**
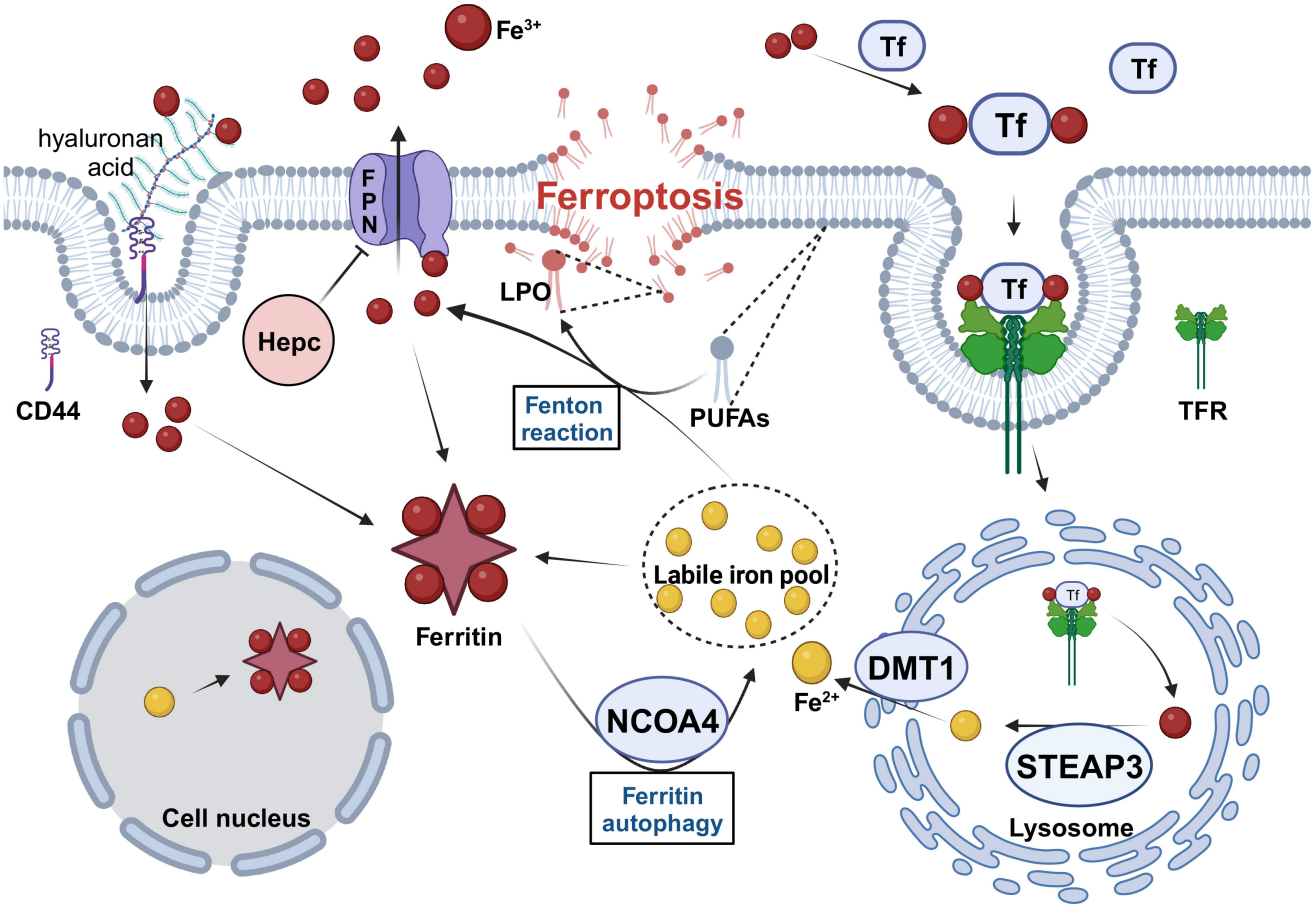
The regulation of iron metabolism in CSCs. CSCs need to maintain high levels of intracellular iron to meet their physiological requirements in four ways. 1. Increase iron uptake by enhancing TF/TFR and CD44-mediated endocytosis. 2. Increase Fe^2+^ levels in the LIP by upregulating the expression of STEAP3, DMT1 and NCOA4. 3. Increase intracellular iron storage and inhibit excessive Fenton reaction by upregulating ferritin expression. 4. Inhibit FPN-mediated iron efflux by downregulating FPN and upregulating Hepc expression. Tf, transferrin; TFR, transferrin receptor; STEAP3, six-transmembrane epithelial antigen of prostate 3; DMT1, divalent metal transporter protein 1; NCOA4, nuclear receptor coactivator 4; FPN, Ferroportin; Hepc, hepcidin; PUFAs, polyunsaturated fatty acids; LPO, lipid hydroperoxide. Created with BioRender.com.

In summary, high iron levels were maintained in CSCs by increasing iron uptake, improving LIP homeostasis, increasing iron stores, and decreasing iron exports (**Figure 1**). However, the mechanism by which these regulatory mechanisms work synergistically to regulate iron homeostasis in CSCs has not yet been thoroughly investigated. Furthermore, it is unclear whether higher iron levels are a causal factor or consequence of CSC production. Iron supplementation has been found to induce stemness and promote EMT in CSCs (9), and it seems that higher iron levels are a causal factor in CSC production. Furthermore, iron chelators can inhibit the stemness and growth of CSCs by reducing the intracellular iron levels (60). However, researchers found that in an iron-deficient environment, CSCs upregulate TFR and DMT1 expression to increase iron uptake and intracellular iron availability, thereby enhancing growth and maintaining CSC stemness (60). In addition, recent studies have shown that ER+ breast cancer cells transform into drug-resistant CSCs after co-culturing with stem cells and increase intracellular levels of Fe^2+^ (61). This appears to be a consequence of the presence of CSCs, which leads to high intracellular iron levels.

However, the increase in iron levels in CSCs is significant and has several important implications. Most of the iron that enters cells is used to synthesize heme and iron-sulfur clusters. These compounds have critical functions in mitochondrial energy metabolism (62), and they each plays a different role. Heme induces the epithelial-mesenchymal transition (EMT) by regulating BTB and CNC homology 1 (BACH1) (63). In addition, it can promote angiogenesis and nerve growth, resulting in a favorable TME (64). Iron-sulfur clusters are involved in the composition of multiple DNA repair enzymes (helicases, nucleases, glycosylases, and demethylases) and ribonucleotide reductase (65). Therefore, an increased number of iron-sulfur clusters is most likely the reason for the strong DNA repair ability of CSCs. On the other hand, as a cofactor of the prolyl/asparagyl hydroxylase (PHD) family, free intracellular iron can stabilize HIF-1α, inducing defective angiogenesis and aerobic glycolysis (62). Mechanistically, increased nuclear iron has been shown to be the rate-limiting condition for epigenetic reprogramming (40), that iron acts as a co-factor of epigenetic enzymes (TET enzymes and JmjC domain-containing proteins), whose mediated epigenetic reprogramming regulates the canonical Notch, Hedgehog, Wnt/GSK-3β/β-catenin, and TGF-β1/Smad2/3 signaling pathways to maintain stemness and self-renewal capacity or induce EMT (66–69).

In conclusion, since iron levels are significantly elevated in CSCs, and this alteration in iron homeostasis facilitates the maintenance of stemness, research into the mechanisms regulating iron homeostasis in CSCs is necessary to discover additional therapeutic approaches that can be used to eradicate CSCs by targeting iron metabolism.

## 3 Lipid peroxidation in CSCs

### 3.1 Mechanism of lipid peroxidation

In the decade since ferroptosis was first named, peroxidation of phospholipids acylated with polyunsaturated fatty acids (PL-PUFAs) in cell membranes to lipid peroxides has been identified as a driver of ferroptosis. Although cholesterol peroxidation (70) and ether lipid synthesis (71) can affect ferroptosis, it has been suggested that lipid peroxidation in ferroptosis is best characterized in the context of phospholipids because the diallyl hydrogens in PL-PUFAs are more susceptible to extraction by strong oxidants and free radical formation than those in saturated or monounsaturated fatty acids, making PL-PUFAs the most susceptible to oxidation (70). Phospholipid peroxidation can be initiated in cells in both non-enzymatic and enzymatic manners. Non-enzymatic lipid peroxidation is mainly catalyzed by iron in LIP, which generates lipid peroxides via the Fenton reaction, whereas enzymatic lipid peroxidation can also generate lipid peroxides, such as the lipoxygenase family (LOXs) (72), NADPH-cytochrome P450 reductase (POR) (73). However, these enzymes are iron-dependent (74).

Although, previous studies have found that the inhibition of 5-LOX downregulates stemness and inhibits the growth of prostate CSCs (75); and that 15-LOX is required for the survival of chronic granulocytic leukemia stem cells (76). It may seem paradoxical that on the one hand LOXs play an important role in maintaining CSCs; however, LOXs can promote lipid peroxidation to induce ferroptosis, as phosphatidylethanolamines (PE-AA and PE-AdA) containing arachidonic acid (AA) and adrenaline (AdA) can be oxidized by LOXs (22, 77). Furthermore, as an antioxidant, vitamin E can prevent ferroptosis by inhibiting LOX-catalyzed phospholipid peroxidation (78), making it difficult to ignore the role of LOX in ferroptosis. Unsurprisingly, some scientists believe that the role of LOX-mediated enzymatic lipid peroxidation in ferroptosis is controversial (22). LOXs promote ferroptosis under certain conditions. Studies have shown that LOXs form a complex with phosphatidylethanolamine-binding protein 1 (PEBP1) to catalyze phospholipid peroxidation in the membrane and that PEBP1 binding to PUFAs is required for LOXs to induce ferroptosis (79).

Similarly, recent studies have shown that POR can increase the peroxidation of PUFAs to promote ferroptosis (80), mainly by acting in conjunction with NADH-cytochrome b5 reductase (CYB5R1), which uses NADPH and O2 as substrates to produce H2O2, and then participates in the iron-mediated Fenton reaction to cause lipid peroxide accumulation, thereby promoting ferroptosis (73). The role of POR in CSCs has not been reported, although one study confirmed that in triple-negative breast cancer, high POR expression was strongly associated with shorter recurrence-free survival (RFS) and did not significantly correlate with overall survival (OS) (81). This also suggests that higher POR expression is associated with earlier recurrence, which contradicts its role in promoting ferroptosis.

The role of iron-mediated non-enzymatic lipid peroxidation in ferroptosis is unclear, and the significant increase in iron levels in CSCs should lead to an increase in lipid peroxidation produced by the iron-mediated Fenton reaction; however, it may not lead to accumulation of lipid peroxidation to the extent of inducing ferroptosis, as CSCs have a robust redox system that resists oxidative stress (82) to avoid ferroptosis (83). It has been suggested that reactive oxygen species (ROS), dependent on the mitochondrial respiratory chain and NADPH oxidase (NOX) activity, promote LOXs- and POR-mediated lipid peroxidation (84). However, the extent to which enzymatic lipid peroxidation plays a role in ferroptosis and its role in ferroptosis is inconclusive. We speculated that to maintain their stemness characteristics and survival, CSCs require high expression of LOXs and POR to perform functions other than lipid peroxidation. The POR plays an important role in various metabolic mechanisms and is involved in the metabolism of steroid hormones, drugs, and xenobiotics (85). However, this suggests that lipid peroxide production in CSCs may be high under the combined action of iron and enzymes.

### 3.2 Phospholipid synthesis increased in CSCs

Lipids are important cellular components and energy sources. Regardless of the cancer origin, CSCs have higher lipid levels than non-CSCs. To maintain stemness characteristics and meet survival needs, CSCs adapt to lipid metabolism by upregulating *de novo* fatty acid synthesis, lipid uptake, lipid desaturation, oxidation, and lipid droplet synthesis, etc. (83, 86). Phospholipids containing PUFAs are most susceptible to peroxidation during ferroptosis. Therefore, this section mainly describes the effects of alterations in lipid metabolism on phospholipids or PUFAs in CSCs and then analyzes and discusses their effects on ferroptosis in CSCs.

#### 3.2.1 Increased *de novo* lipogenesis in CSCs

Fatty acid synthesis is more active in CSCs than in non-CSCs, and its key enzymes are expressed at higher levels in CSCs, such as ATP citrate lyase (ACLY), acetyl coenzyme A carboxylase (ACC), fatty acid synthase (FASN), and sterol regulatory element-binding protein (SREBP), which regulate the expression of these genes (86). FASN-mediated fatty acid synthesis promotes gemcitabine resistance in pancreatic cancer cells, whereas the FASN inhibitor, orlistat, reduces pancreatic cancer cell stemness (87). The inhibition of fatty acid synthesis has also been shown to inhibit CSC growth in glioma stem cells and breast CSCs both *in vivo* and *in vitro* (88, 89). Because saturated fatty acids (SFAs) are the products of fatty acid synthesis, it is surprising that the upregulation of fatty acid synthesis in non-CSCs increases the susceptibility of cells to ferroptosis. In several cell models, FASN promotes ferroptosis by inhibiting the SLC7A11-GPX4 axis to reduce lipid peroxide clearance, and ACC1 sensitizes cells to ferroptosis by promoting the peroxidation of PUFAs (70). In CSCs, key enzymes involved in fatty acid synthesis, including FASN and ACC1, are upregulated, which appear to increase the accumulation of lipid peroxides in cells. This may be due to the strong lipid peroxidation-scavenging ability of CSCs in avoiding ferroptosis.

#### 3.2.2 Enhanced lipid uptake in CSCs

Studies have shown that ovarian cancer cells undergo reprogramming of lipid metabolism during the development of resistance to platinum and that the increase in intracellular lipids is dominated by lipid uptake rather than fatty acid synthesis (90), suggesting that lipid uptake is important for the development of CSCs. Alterations in lipid metabolism have been reported in CSCs, including increased lipid uptake (10) and expression of CD36, which promotes EMT by increasing extracellular lipid uptake (91). However, the expression levels of fatty acid transporters (such as CD36 and FATP3) in mesenchymal gastric cancer cells (GCs) were not significantly different from those in enteric GCs, and FATP2 expression levels were lower than those in enteric GCs (92, 93). Therefore, not all CSCs showed an increased uptake of extracellular lipids, which we believe may be related to the energy metabolism of CSCs. Many studies have confirmed that CSCs can select different energy metabolism mechanisms according to different tumor microenvironments, which may be oxidative phosphorylation (OXPHOS), glycolysis or β-oxidation (10). This also reflects the high metabolic heterogeneity and plasticity of CSCs; that is, they can metabolize energy using the most efficient mechanisms to meet their own needs. However, our view needs to be confirmed by further research.

#### 3.2.3 Increased lipid desaturation in CSCs

Human cells cannot synthesize PUFAs endogenously and can only take up 18-carbon PUFAs, such as linoleic acid (LA) and alpha-linoleic acid (ALA), from the environment, which are subjected to ELOVL fatty acid elongase 5 (ELOVL5) and fatty acid desaturase1/2 (SCD1/FADS2), respectively (10). Finally, they are synthesized into more complex lipids, such as diacylglycerides (DAGs) and triacylglycerides (TAGs), or converted into phosphoglycerides, such as phosphatidic acid (PA), phosphatidylethanolamine (PE), and phosphatidylserine (PS) (92). Lipid unsaturation is increased (94) and upregulation of ELOVL5 and SCD1/FADS2 expression is often found in tumors with high aggressiveness and drug resistance and contributes to maintaining the stemness and aggressiveness of malignant tumors (95). Because cell membrane fluidity is determined by lipid unsaturation (96), reducing membrane fluidity inhibits the metastatic and stemness characteristics of breast cancer (97). Therefore, lipid desaturation is critical in CSCs. In addition, SCD1 converts SFAs from lipid uptake and *de novo* synthesis into MUFA, thus preventing SFAS-induced lipotoxicity while contributing to cell survival (98). MUFA are now known to play a role in preventing or limiting ferroptosis (70). Lipid desaturation plays a dual role in CSC ferroptosis. One is to increase the amount of intracellular PUFAs, which increases the susceptibility to ferroptosis, and the other is to increase the synthesis of MUFAs to protect cells from ferroptosis. FADS2 knockdown reduces the abundance of intracellular PUFAs, resulting in decreased sensitivity to ferroptosis (99), whereas SCD1 knockdown increases the sensitivity of ovarian CSCs to ferroptosis by reducing the synthesis of cytoprotective lipids (MUFAs) (100).

#### 3.2.4 Enhanced β-oxidation in CSCs

Owing to their high metabolic flexibility, CSCs can induce fatty acid oxidation to ensure their survival in the absence of glucose (83). For example, the stemness marker NANOG can reduce mitochondrial ROS production by inhibiting OXPHOS, while increasing intramitochondrial fatty acid oxidation, thereby maintaining self-renewal and drug resistance in CSCs (101). Previous studies have shown that mitochondrial ROS do not contribute to ferroptosis (21). However, we believe that reducing mitochondrial ROS production is important for maintaining intracellular REDOX homeostasis, and may indirectly reduce the susceptibility of CSCs to ferroptosis. Furthermore, mitochondrial membranes undergo significant changes during ferroptosis, and more importantly, more than 10% of the cellular GSH is present in the mitochondria (102), making it difficult to argue that mitochondrial ROS has no effect on ferroptosis. Several studies have found that fatty acid oxidation maintains the stemness profile and drug resistance of breast CSCs (103), leukemia stem cells (LSCs) (104), and mesenchymal GCs (105) and possibly through increased fatty acid oxidation of more metabolic intermediates (e.g., acetyl coenzyme A and NADH) (106). However, a study with opposite results found reduced expression of peroxisome proliferator-activated receptor γ (PPARγ) in hepatic CSCs. As PPARγ regulates the expression of many genes involved in fatty acid oxidation, the researchers concluded that fatty acid β-oxidation is inhibited in hepatic CSCs (107). We believe that this reflects the flexibility of CSCs metabolism; however, its specific effect on ferroptosis is unknown.

#### 3.2.5 Increased lipid droplets in CSCs

Lipid droplets (LDs) are dynamic and functional organelles with an outer monolayer of phospholipids that store neutral lipids, such as triacylglycerides (TAGs), cholesteryl esters, and retinol, and an increased number of lipid droplets is associated with high tumor aggressiveness and drug resistance (83, 108). A significant increase in LDs has been reported in colon, breast, ovarian, and prostate CSCs (10). Functionally, LDs not only maintain membrane and ER homeostasis by translocating potentially toxic lipids to the inner LD to isolate and tightly regulate lipid synthesis and catabolism but also actively respond to the need for fatty acid oxidation within the mitochondria (109). More importantly, during oxidative stress, LDs inhibit PUFA oxidation by translocating PUFAs from the membrane to the LD core to protect neuroblastoma cells (110). In ovarian CSCs, researchers have found higher LD content in CSCs with a higher proportion of unsaturated lipids (94). LDs play an important protective role in CSCs against ferroptosis and other cytotoxic effects (83).

### 3.3 Phospholipid remodeling enhanced in CSCs

Regardless of the extent of intracellular accumulation of Fe^2+^ and lipid peroxides, ferroptosis is ultimately reflected in the disruption of membrane stability, which includes not only the plasma membrane, but also the membranes of organelles such as mitochondria, lysosomes, and the endoplasmic reticulum (39, 111, 112). The most significant changes in cell morphology were observed in the mitochondria. Upon ferroptosis, mitochondria decrease in volume, increase in membrane density, experience a reduction or disappearance of cristae, and undergo rupture of the outer membranes (21). Phospholipid remodeling is particularly important for maintaining membrane homeostasis.

Glycerophospholipids (GPLs) are essential components of the cytoplasmic membrane that are produced by cells *via de novo* synthesis and lipid uptake. During the remodeling of GPLs (also known as the Lands cycle), phospholipase A2 (PLA2) cleaves oxidized PUFAs at the sn-2 position, whereas SFAs and MUFAs are acylated at the sn-1 and sn-2 positions of GPLs, limiting the accumulation and spread of lipid peroxides across the membrane, thereby reducing the susceptibility to ferroptosis (70). Subsequently, lysophospholipids (LPLs) re-acylate PUFAs at the sn-2 position, catalyzed by lysophosphatidylcholine acyltransferase 3 (LPCAT3) (113). It was also easy to identify the inhibitory effect of PLA2 on ferroptosis and the ferroptosis-promoting effect of LPCAT3. PLA2 is highly expressed in pancreatic, hepatocellular, breast, colon, and prostate cancers and is strongly associated with poor prognosis (113). In contrast, knockdown of LPCAT3 allows cells to avoid ferroptosis (70).

Long-chain acyl-CoA synthetase (ACSL) is also important for phospholipid remodelling, with ASCL3 and ASCL4 specifically activating MUFAs and PUFAs, respectively, prior to remodelling, both of which have been shown to influence cellular sensitivity to ferroptosis by regulating the proportion of MUFAs and PUFAs in membrane PLs (114). Thus, phospholipids containing MUFAs protect cells from ferroptosis, whereas phospholipids containing PUFAs are highly susceptible to peroxidation, thereby promoting ferroptosis (70). Thus, it is clear that ACSL3 inhibits ferroptosis by increasing membrane stability through its involvement in the remodelling of PL-MUFAs, and that high expression of ACSL3 in human melanoma is associated with poorer prognosis (115). In contrast, ACSL4 overexpression sensitizes breast cancer cells to ferroptosis (116). The ACSL4 knockout has also been shown to inhibit ferroptosis (70). Interestingly, increased ACSL4 expression is associated with increased aggressiveness and drug resistance in breast and prostate cancers (117). Thus, we suggest that ACSL4 exerts different effects on ferroptosis under different conditions. ACSL1 can act as either a promoter or an inhibitor of ferroptosis under different conditions (70).

In summary, there are two main aspects of lipid peroxidation in the CSCs. First, the expression levels of enzymes involved in the mechanism of enzymatic lipid peroxidation are upregulated in CSCs; although the extent to which these enzymes play a role is not yet fully understood, their role cannot be ignored. Furthermore, as the levels of Fe^2+^ in the LIP in CSCs increase, their mediated non-enzymatic lipid peroxidation is also enhanced. Therefore, the mechanism of lipid peroxidation is enhanced in CSCs. The second mechanism involves the synthesis and remodelling of phospholipids, which are substrates for lipid peroxidation. Although the mechanisms of lipid metabolism in CSCs are flexible and variable in all aspects to facilitate their survival in a hostile environment, the end result is an increase in intracellular lipid content and lipid unsaturation. Therefore, under the action of these two factors, the production of intracellular lipid peroxidation also increases, which is consistent with the finding in many studies that CSCs are more sensitive to ferroptosis (72, 114) and appear to have a strong lipid peroxide scavenging capacity.

## 4 Increased lipid peroxide scavenging capacity in CSCs

Lipid peroxidation in membranes has been reported to significantly alter physiological roles such as membrane permeability, membrane fluidity, ionic gradients, and signaling pathways (118). As mentioned above, CSCs are characterized by high ferrous levels, high lipid levels, and high lipid unsaturation; however, the consequences triggered by these characteristics seem to point to an increase in intracellular lipid peroxidation, which plays an important role for CSCs in tumor recurrence, metastasis, and drug resistance. Furthermore, ROS are maintained at lower levels compared to non-CSCs (119), particularly during the quiescent phase (14). This suggests that CSCs have a powerful redox system that can scavenge peroxidized lipids in a timely and effective manner to maintain membrane stability and avoid ferroptosis. It has been reported that a robust antioxidant and ROS scavenging system may not only reduce basal ROS levels in colon CSCs but also promote drug resistance by preventing lethal ROS elevation during drug treatment (14). Among the antioxidant defense networks present in the cell, the main mechanisms of lipid peroxidation scavenging are the GPX4-GSH axis and ferroptosis suppressor protein 1-coenzyme Q10 (FSP1-CoQ10) axes, which are the main defense strategies during ferroptosis.

### 4.1 GPX4-GSH axis activity increased in CSCs

GPX4 is the main enzyme that protects cells from ferroptosis (120). It uses GSH as a substrate to convert lipid peroxides into nontoxic lipid alcohols and produces oxidized glutathione molecules (GSSG) to reduce the intracellular accumulation of lipid peroxides and prevent ferroptosis (121, 122). GSH depletion directly affects the activity and stability of GPX4, thereby promoting cellular ferroptosis (122). GSH is a major intracellular antioxidant composed of cysteine, glutamate, and glycine (123). SLC7A11 (also known as xCT) is a cystine/glutamate retrotransport protein expressed in the plasma membrane that imports extracellular cystine into the cell, which is then reduced to cysteine for GSH synthesis (102). The rate-limiting step in GSH synthesis is catalyzed by glutamate cysteine ligase (GCL) and glutathione synthetase (GSS) (124). GCL is a heterodimeric protein consisting of a catalytic subunit (GCLC) and modifying subunit (GCLM) expressed by different genes (125). Studies have shown that downregulation of GCLC expression can lead to GSH depletion and susceptibility to oxidative stress in a mouse model of liver cancer (126). In addition, the expression of ChaC glutathione-specific gamma-glutamyl-cyclotransferase 1 (CHAC1), which degrades GSH, can effectively induce ferroptosis in hepatoma cells (127).

In a previous study, we suggested that CSCs have a strong peroxidative lipid scavenging capacity and that the GPX4-GSH axis, a major mechanism of peroxidative lipid scavenging, is upregulated in CSCs and contributes to the acquisition and maintenance of stemness characteristics (15). A growing number of studies have shown that the GPX4-GSH axis plays an important antioxidant role in promoting or maintaining the stemness profile and drug resistance of colorectal, gastric, breast, pancreatic, liver, biliary, lung, and glioma CSCs (27, 34, 128–133). In addition, the stem genes *CD133* (134), *CD44* (6), *SOX2* (135), *KLF4* (131), *YAP/TAZ* (130), and *DKK1* (136) directly and/or indirectly (via nuclear factor erythroid-2-related factor 2 (NRF2)) upregulate the GPX4-GSH axis to enhance lipid peroxide scavenging by CSCs to prevent ferroptosis. NRF2 plays a key role in this process. It has been reported that NRF2 expression is upregulated in both CSCs and CSC models (15, 134), that activation of the NRF2 pathway promotes tumorigenicity and stemness in CSCs (137), and that silencing NRF2 inhibits the spherogenic ability of colon CSCs and the expression of markers of stemness (134). More importantly, the activation of NRF2 signaling leads to the maintenance of low ROS levels in CSCs (15). It is now known that NRF2 can upregulate the expression of SCL7A11, GPX4, GCLC, and GSS, and promote the synthesis of GSH and the scavenging of lipid peroxidation. This finding demonstrates the importance of the cellular redox system involving NRF2 in CSCs.

Given the importance of GPX4-GSH in scavenging lipid peroxides, many researchers have attempted to promote ferroptosis by targeting this system to eradicate CSCs. For example, some researchers have used dihydroartemisinin (DHA) to downregulate GPX4 expression, leading to intracellular lipid peroxide accumulation and ferroptosis promotion in glioblastoma (138). Other studies have targeted xCT using salazosulfapyridine and protein kinase C alpha (PKCα) inhibitors to reduce intracellular GSH levels and promote ferroptosis in neuroblastoma stem cells (139).

### 4.2 FSP1-CoQ10 axis activity increased in CSCs

FSP1 was originally known as apoptosis-inducing factor mitochondria-associated 2 (AIFM2), it was later found that FSP1 can also remove lipid peroxidation and prevent ferroptosis in the absence of GPX4 (140). The inhibitory effect of FSP1 on ferroptosis is mediated by CoQ10: reductive CoQ10H2 captures and removes free radicals, and FSP1 uses NADH/NADPH to reduce CoQ10 to CoQ10H2 (70, 140). Recent studies have shown a significant upregulation of the protein levels ACSL1 in highly metastatic ovarian cancer cell lines. This, in turn, increases the protein levels FSP1 by blocking protein degradation. Thus, the FSP1-CoQ10 axis is activated, causing iron death resistance, and elevating cancer cell spheroidogenesis and drug resistance. (141). Triple-negative breast cancer (TNBC) displays the breast cancer stem cell (BCSC) phenotype of CD44+/CD24-, possesses tumor-initiating properties, and is associated with stem cell-like characteristics in breast cancer (142). This study revealed that enhanced FSP1-mediated ubiquinone redox metabolism in TNBC inhibited ferroptosis, whereas disruption of the FSP1/CoQ10 axis overcame ferroptosis resistance and inhibited TNBC (143). FSP1 is a downstream effector of NRF2 in lung cancer cells. Moreover, NRF2 has been shown to inhibit ferroptosis through the FSP1-CoQ10 axis, which leads to cancer cell radioresistance. (144) As previously mentioned, NRF2 expression is upregulated in CSCs. Therefore, it is worth considering whether the FSP1-CoQ10 axis is similarly upregulated in CSCs. Therefore, further studies are required.

Previous studies have shown that the GPX4-GSH axis is the primary mechanism for preventing ferroptosis in cells. Dihydroorotate dehydrogenase (DHODH) and GTP cycle hydrolase 1 (GCH1)/tetrahydrobiopterin (BH4), independent of the GPX4-GSH axis, scavenge lipid peroxides and inhibit ferroptosis (145). However, only a few studies have drawn convincing conclusions. In summary, the GPX4-GSH axis and FSP1-CoQ10 axes were significantly upregulated in CSCs and played important antioxidant roles in inducing and maintaining the stemness properties of CSCs.

## 5 Efforts to develop drugs to induce ferroptosis in CSCs

In recent years, promising results have been achieved in the development of drugs that induce ferroptosis in CSCs (**Table 1**). This was reflected in two main areas. The first step is the development of new drugs. Nanocarriers have been proven to be an effective means of targeting CSCs. Researchers have coupled salinomycin with biocompatible polyethylene glycol-coated gold nanoparticles (AuNPs) to improve their specificity for breast CSCs and induce ferroptosis, thereby targeting CSC elimination (156). Drugs loaded onto nanocarriers are flexible and diverse. Researchers developed a GSH-bioimprinted nanoparticle-loaded drug, GNPIPP12MA, which targets leukemic stem cells and induces ferroptosis by depleting intracellular GSH, thereby enhancing the efficacy of chemotherapy and immunotherapy (146). Other researchers have designed hyaluronic acid-encapsulated iron oxyhydroxide-based nanosystems that target breast CSCs and inhibit their proliferation by increasing GSH depletion, Fe^2+^ levels, and iron efflux to increase ferroptosis. The second is the new use of old drugs. For example, drugs, such as ibuprofen, phenazine derivatives, itraconazole, and dihydroartemisinin, promote ferroptosis to kill CSCs or increase their sensitivity to antitumor drugs (39, 138, 150, 153, 155). Since no CSC-targeted ferroptosis-inducing drugs have been approved for clinical use, these older drugs seem to be the closest to clinical application in terms of strategies to induce ferroptosis in CSCs; however, they also affect normal cells indiscriminately while inducing ferroptosis in CSCs (157), so the side effects of these drugs need to be considered in their application. Nanodrugs, although more targeted, face a similar problem of reducing side effects while ensuring efficacy; therefore, there is still a long way to go in the development of new drugs, but also a lot of hope.

**Table 1:** Summary of ferroptosis-based drugs for CSCs therapy.

Name	Target	Mechanism	Type of cancer	References
GNPIPP12MA	GSH	Targeting the FTO/m6A pathway to deplete intracellular GSH	Leukemic stem cells	(146)
Ferroptotic polymer micelles (RSL3)	GPX4	RSL3 targets and inhibits GPX4	Drug-resistant persistent ovarian cancer cells	(147)
Disulfiram	xCT GPX4	Downregulation of xCT and GPX4 expression	Glioblastoma cells	(148)
ALZ003	GPX4	Decreasing GPX4 expression	Glioblastoma cells	(149)
Ibuprofen	Nrf2 xCT GPX4	Downregulation of Nrf2 signaling pathway	Glioblastoma cells	(150)
FeOOH/siPROM2@HA	Prominin2 GPX4	Inhibiting Fe^3+^ efflux and Fe^3+^ consumes GSH to generate Fe^2+^	Breast CSCs	(151)
AuNP-PHF	Ferritin	Inducing ferritin degradation	Breast CSCs	(152)
Phenazine derivatives	Iron	Sequestering iron in lysosomes	Breast CSCs	(153)
Dichloroacetate	Iron	Sequestering iron in lysosomes	Colorectal CSCs	(112)
Atranorin@SPION	GPX44 SLC24A4	Inducing ferritin degradation	Gastric CSCs	(154)
Itraconazole	Iron	Sequestering iron in lysosomes	Nasopharyngeal CSCs	(155)
Dihydroartemisi-nin	GPX4	Downregulation of GPX4	Glioblastoma cells	(138)
Sal-AuNPs	Iron GPX4	Iron accumulation and inhibition of antioxidant properties	Breast CSCs	(156)
Salinomycin	DMT1	Blocking lysosomal iron translocation	Breast CSCs	(45)
Inhibitor of PKCα (Sulfasalazine, C2-4)	GPX4	Downregulating GPX4 activity and stimulating lipid peroxidation	Neuroblastoma CSCs	(139)

## 6 Summary and outlook

Drug resistance, relapse, and metastasis have always plagued the treatment of tumors, and as research has progressed, we have become increasingly aware of the important role of CSCs. Therefore, there is an urgent need for more effective strategies to eradicate CSCs and improve the prognosis of patients with tumors. Over the past decade, considerable progress has been made in the study of ferroptosis, which has raised hopes for its potential as an antitumor therapy, but what we can do therapeutically still seems limited with no significant breakthrough in clinical application. Ferroptosis is cell death caused by an imbalance in the production and clearance of lipid peroxides mediated by Fe^2+^(158). Many studies have been conducted to elucidate the morphological changes, biochemical manifestations, lipid peroxidation mechanisms, and redox regulation mechanisms of ferroptosis; however, there are still many issues that require further investigation. For example, what is the role of the mitochondria in ferroptosis? Why are the mechanisms of action of certain enzymes involved in ferroptosis contradictory? At what point does lipid peroxide accumulate before completely disrupting membrane homeostasis? And to what extent do other cellular antioxidant systems play a role in ferroptosis since, after all, the synthesis requires NADPH. In addition, given that the mechanism for inducing ferroptosis in normal and tumor cells is flexible and changeable, and that there are many genes and metabolic mechanisms involved in ferroptosis, self-renewal of CSCs is more flexible in dealing with ferroptosis.

Despite the many difficulties, an increasing number of researchers are focusing on the eradication of CSCs by ferroptosis to achieve the desired therapeutic effects. From the above analysis and summary, it can be seen that to maintain stemness and self-metabolism, CSCs maintain higher levels of iron, Fe^2+^, lipids, and unsaturated lipids in cells than non-CSCs, and they should also be subjected to more iron-mediated unsaturated lipid peroxidation, which may be the vulnerability of CSCs. Although CSCs have a stronger antioxidant system, they appear to have reached the upper limit of the threshold that cells can withstand, and many studies have found that CSCs, mesenchymal tumor cells, and drug-resistant tumor cells are more sensitive to ferroptosis (34, 124, 159). CSCs are also more susceptible to ferroptosis than apoptosis (160). It is as if CSCs are “daring” to dance on the knife edge of ferroptosis, which is more exciting but also more dangerous. However, this suggests that eradicating CSCs by inducing ferroptosis is likely to be an effective antitumor strategy.

In conclusion, given the great potential and application of therapeutic strategies targeting CSCs through ferroptosis induction in tumor treatment, further research on CSCs and ferroptosis is needed to develop effective antitumor drugs targeting CSCs.

## Author contributions

HW explored the topic, defined the formation, and drafted the manuscript. ZZ and SR participated in writing and editing the manuscript critically. QY, YC, and JC contributed to design search strategies, to retrieve papers, and to prepare the images. BH, SH, and XW revised the manuscript and approved of the final version. All authors contributed to the article and approved the submitted version.
